# The Association of Multiple Gene Variants with Ageing Skeletal Muscle Phenotypes in Elderly Women

**DOI:** 10.3390/genes11121459

**Published:** 2020-12-05

**Authors:** Praval Khanal, Lingxiao He, Adam J. Herbert, Georgina K. Stebbings, Gladys L. Onambele-Pearson, Hans Degens, Christopher I. Morse, Martine Thomis, Alun G. Williams

**Affiliations:** 1 Musculoskeletal Science and Sports Medicine Research Centre, Department of Sport and Exercise Sciences, Manchester Metropolitan University, Manchester M15 6BH, UK; lingxiao.he@hotmail.com (L.H.); g.stebbings@mmu.ac.uk (G.K.S.); g.pearson@mmu.ac.uk (G.L.O.-P.); c.morse@mmu.ac.uk (C.I.M.); a.g.williams@mmu.ac.uk (A.G.W.); 2 Department of Movement Sciences, Physical Activity, Sports & Health Research Group, KU Leuven, 3001 Leuven, Belgium; martine.thomis@kuleuven.be; 3 Department of Sport and Exercise, Birmingham City University, Birmingham B5 5JU, UK; adam.herbert@bcu.ac.uk; 4 Department of Life Sciences, Manchester Metropolitan University, Manchester M15 6BH, UK; h.degens@mmu.ac.uk; 5 Institute of Sport Science and Innovations, Lithuanian Sports University, LT-44221 Kaunsas, Lithuania; 6 Pharmacy of Targu Mures, University of Medicine, 540142 Targu Mures, Romania; 7 Institute of Sport, Exercise and Health, University College London, London W1T 7HA, UK

**Keywords:** single nucleotide polymorphisms, neuromuscular, elderly, genotyping

## Abstract

There is a scarcity of studies that have investigated the role of multiple single nucleotide polymorphisms (SNPs) on a range of muscle phenotypes in an elderly population. The present study investigated the possible association of 24 SNPs with skeletal muscle phenotypes in 307 elderly Caucasian women (aged 60–91 years, 66.3 ± 11.3 kg). Skeletal muscle phenotypes included biceps brachii thickness, vastus lateralis cross-sectional areas, maximal hand grip strength, isometric knee extension and elbow flexion torque. Genotyping for 24 SNPs, chosen on their skeletal muscle structural or functional links, was conducted on DNA extracted from blood or saliva. Of the 24 SNPs, 10 were associated with at least one skeletal muscle phenotype. *HIF1A* rs11549465 was associated with three skeletal muscle phenotypes and *PTK2* rs7460 and *ACVR1B* rs10783485 were each associated with two phenotypes. *PTK2* rs7843014, *COL1A1* rs1800012, *CNTF* rs1800169, *NOS3* rs1799983, *MSTN* rs1805086, *TRHR* rs7832552 and *FTO* rs9939609 were each associated with one. Elderly women possessing favourable genotypes were 3.6–13.2% stronger and had 4.6–14.7% larger muscle than those with less favourable genotypes. These associations, together with future work involving a broader range of SNPs, may help identify individuals at particular risk of an age-associated loss of independence.

## 1. Introduction

Ageing is a complex physiological process and is associated with a decline in skeletal muscle function [1]. Neuromuscular function determines an individual’s mobility and independence during old age [2,3]. The heritability values of muscle mass and muscle strength are reported to be between 45–82%, depending on the skeletal muscle phenotypes and the population considered [4,5,6], which suggests that associations between single nucleotide polymorphisms (SNPs) and skeletal muscle phenotypes could, in aggregate, account for a substantial portion of the typical inter-individual variability in skeletal muscle mass and strength. Genotype affects skeletal muscle in several ways, influencing myoblast proliferation, enhancing the transcription of muscle-specific genes, mitochondrial function and the activation of signalling pathways [7,8,9], all of which play a role in the maintenance of muscle mass and muscle function [10,11].

There is considerable inter-individual variability in muscle size and muscle strength, with up to 18% [12] and 20% [13] population variability reported for appendicular lean muscle size and vastus lateralis (VL) muscle volume respectively, and up to 16% coefficient of variation for specific force [13,14] in younger adults. Assuming all else is equal, this variance implies that within the elderly population, those at the weaker or lower end of this distribution are more likely to experience a loss of independence at an earlier age. To date, there are numerous studies associating single SNPs with skeletal muscle phenotypes in a variety of populations, ranging from young adult athletes to elderly members of the general population [15,16]. The outcome of such studies is, however, equivocal, as there are instances where these same SNPs show contrasting results depending on the population investigated. In older adult populations, for instance, *ACE* insertion/deletion (*ACE* I/D) is associated with skeletal muscle mass phenotypes [10] in one study, whereas in another study they are not associated [17].

When selecting meaningful phenotypes to investigate for possible associations with SNPs, it is important to consider skeletal muscle phenotypes that are relevant to health-related quality of life (HQoL) and activities of daily living (ADL). For instance, vastus lateralis (VL) muscle atrophy is representative of muscle loss associated with ageing [18] and lower muscle mass has been linked with functional impairment and physical disability in older people [19]. Similarly, loss of knee extensor strength correlates with functional impairments in the elderly [20]. In addition to lower limb musculature, upper limb muscle size and muscle strength are also prone to decline with ageing [21,22] and low hand grip strength (HGS) has been previously linked with impaired mobility, functional decline and higher levels of mortality [23,24,25]. The identification of new gene variants or replicating previous findings in an elderly population could be useful in identifying elderly people at a particularly enhanced risk of mobility limitations.

Despite equivocal single-SNP associations being reported [10,17,26,27], there have been no investigations of multiple in vivo skeletal muscle size and strength measures in elderly women for associations with multiple gene variants. Therefore, the present study investigated the association of a selection of 24 SNPs with skeletal muscle phenotypes, specifically muscle size (biceps brachii thickness and vastus lateralis anatomical cross sectional area (VL_ACSA_)) and muscle strength (handgrip strength (HGS), isometric elbow flexion torque (MVC_EF_) and isometric knee extension torque (MVC_KE_)) measures, in elderly Caucasian women.

## 2. Materials and Methods

### 2.1. Participants

Three hundred and seven Caucasian women aged 60–91 years old (70.7 ± 5.7 years, 66.3 ± 11.3 kg, 1.60 ± 0.06 m) (mean ± SD) who were ambulatory and had no history of severe muscle, bone, nervous system or cardiovascular related disorders, such as osteoporosis, rheumatoid arthritis, cancer, Alzheimer’s, convulsions and epilepsy, volunteered for this study. All procedures were in accordance with the ethical standards of the institution research committee (Manchester Metropolitan University Ethics Committee; Approval number: 09.02.16 (i)) and with the Declaration of Helsinki. Informed consent was obtained from all participants.

Testing was conducted in one session in the following order: anthropometry, handgrip strength, isometric knee extension maximum voluntary contraction (MVC_KE_) and isometric elbow flexion maximum voluntary contraction (MVC_EF_), ultrasound of biceps brachii and vastus lateralis muscle and sample collection (blood or saliva). DNA extraction and genotyping were performed later. The detail of these procedures can be seen in our published paper [28]; however, a brief description of procedures is given below.

### 2.2. Handgrip Strength

A digital load cell handgrip dynamometer (JAMAR Plus, JLW Instruments, Chicago, IL, USA) was used to measure the handgrip strength of the participants [29]. In short, participants performed this test in a standing position, holding the dynamometer with the arm flexed at 90° to the shoulder. During the test, the participants squeezed with maximum effort. The left and right arms were alternated; three trials were performed with each arm. Peak grip strength of all the trials was recorded for the study. The test–retest reliability of measuring HGS with this method is reported to be high (ICC = 0.99) [30].

### 2.3. Isometric Knee Extension and Elbow Flexion Maximal Voluntary Contraction

Isometric knee extension maximal voluntary contraction (MVC_KE_) was recorded using a load cell (Zemic, Eten-Leur, The Netherlands) with all participants in a seated position in a custom-built dynamometer with knee angle maintained at 120° (straight was considered as 180°). The load cell was calibrated prior to every strength testing session. The participants were asked for their dominant side and the dominant leg was securely fastened above the lateral malleolus (identified by palpation) where the participant felt comfortable while fastening the strap (low compliance nylon attached to a force transducer). Participants were instructed to perform MVC_KE_ with real-time visual feedback and verbal encouragement. Three trials were performed, with breaks of 1 min between trials to reduce any influence of fatigue [31]. The force produced was digitized using an analogue-to-digital converter, displayed and recorded on a PC (My LabVIEW, National Instruments, Berkshire, UK). MVC_KE_ was calculated as knee torque considering the length of the tibia, height of the strap and angle of knee extension above the ankle joint in N·m.

MVC_KE_ = Force × (Tibia length − strap distance from ankle) × cos 30°

with the same equipment, MVC_EF_ was performed at 60° elbow flexion (0° is a straight position) and MVC_EF_ was calculated as MVC_EF_ torque (N·m) as

MVC_EF_ = Force × Radius length × cos 30°

### 2.4. Biceps Brachii Thickness

B-mode ultrasonography (My LabTwice, Esaote Biomedical, Genoa, Italy) with a 38-mm probe (7.5 MHz, linear array) was used to measure biceps brachii thickness, following a previously established method [32]. Participants were asked to identify their dominant side and were seated with the dominant arm hanging relaxed at their side; the proximal (acromion process) and distal (olecranon) ends of the humerus were identified using ultrasound scanning and palpation. Thereafter, a sagittal plane scan was performed at 60% length from the proximal end of the humerus, identifying the upper and lower aponeurosis of the biceps brachii muscle [33]. Minimal pressure (denoted by no indentation of the tissue within the field of vision) was applied to the ultrasound probe while scanning to avoid compression of the muscle. The ultrasound was recorded in real time, from which an image was captured and biceps brachii thickness was measured as the distance between the superficial and deep aponeurosis, taken at the proximal, middle and distal end of the captured image using digitizing software (ImageJ 1.45, National Institutes of Health, Bethesda, USA). The mean of the three measurements was recorded as the biceps brachii thickness.

### 2.5. Vastus Lateralis Muscle Area

B-mode ultrasonography (My LabTwice, Esaote Biomedical, Genoa, Italy) was used to determine vastus lateralis (VL) muscle area. With participants standing, the origin and insertion of the VL muscle was identified as the proximal and distal myotendinous junction of the VL, respectively, using ultrasound (7.5 MHz, linear array probe, 38 mm). The origin and insertion of the VL were assessed in a standing position, as the accumulation of subcutaneous fat in some participants made identification of the VL origin impossible in the supine position. The VL length was measured with a measuring tape as the distance from origin (head of femur) to insertion (VL myotendinous junction). The lateral and medial borders of the VL were identified using ultrasound to identify the mid-sagittal line of the VL. Participants were then seated for subsequent ultrasound procedures.

For vastus lateralis anatomical cross-sectional area (VL_ACSA_), a transverse plane ultrasound scan was performed at 50% of VL length, as this corresponds to the VL length at which maximum ACSA is found [34]. Using echo absorptive markers every 3 cm from the medial to the lateral border of the VL muscle, the ultrasound probe was steadily moved over the echo-absorptive markers from the medial to the lateral edge of the VL. The ultrasound was recorded as a digital video file, from which individual images were acquired using capture software. Captured images were acquired at contiguous intervals between each shadow cast by the echo-absorptive markers. The entire VL_ACSA_ was reconstructed in a single canvas from each captured image. For the measurement, digitizing software (ImageJ 1.45, National Institutes of Health, Bethesda, USA) was used as the visible aponeurosis around the VL. The reliability and validity of this method were previously reported to be high (>0.99) when compared with MRI [35].

### 2.6. SNP Selection

For the present study, 24 SNPs were selected. Those SNPs were selected based on several criteria, such as their previous associations with skeletal muscle phenotypes in different populations, their known physiological (functional) mechanism for possible association and some novel gene variants that could influence skeletal muscle phenotypes, as previously reported, to influence other similar phenotypes. While selecting the candidate gene variants from the list of SNPs, priority was given to the frequency of extant literature for SNPs and muscle phenotypes, the presence of conflicting results for SNPs and the already-known transcriptional differences for some of the SNPs. The list of selected gene variants and their previous associations with similar phenotypes is presented in Table S1.

### 2.7. Sample Collection, DNA Extraction and Genotyping

Biological samples were collected as either a venous blood or saliva sample. Briefly, 5 mL of blood was collected from a superficial forearm vein by a trained phlebotomist into 5 mL EDTA tubes (BD Vacutainer Systems, Plymouth, UK). Samples were stored at −20 °C before further processing. Saliva samples were collected using Oragene DNA OG-500 collection tubes (DNA Genotek Inc., ON, Canada) following the manufacturer’s instructions and stored at room temperature until DNA extraction. Genomic DNA was extracted from the collected samples using a QIAcube, QIAamp DNA Blood Mini kit and standard spin column protocol (Qiagen, Crawley, UK). For genotyping, two techniques were used; EP1 Fluidigm (Fluidigm, Cambridge, UK) and StepOnePlus (Applied Biosystems^®^, Paisley, Scotland, UK). A brief description of genotyping procedures using both techniques is presented in our previous papers [36,37] and the genotypes for the selected SNPs were called based on end-point fluorescence (https://www.thermofisher.com/np/en/home.html) (attached in Table S2). All samples were analysed in duplicate [38].

### 2.8. Statistical Analysis

The frequency of all the selected polymorphisms was checked for compliance with the Hardy–Weinberg equilibrium using chi-square tests. Analysis of covariance (ANCOVA) was used to test any genotype effects on skeletal muscle phenotypes (muscle size, muscle strength) with age used as a covariate. When too few participants were in one homozygous group, this group was combined with the heterozygous group and a two-group analysis was performed. Similarly, when there was an association (*p* < 0.05) or a tendency for an association (0.05 < *p* < 0.15) [39], the homozygous and heterozygous groups with closer means were combined and then ANCOVA was re-run for the analysis. All significant associations identified in the main ANCOVA analyses were subject to the Benjamini–Hochberg correction [40,41] with a 20% false discovery rate considering two families (muscle size and muscle strength) of 24 tests each. All statistical analyses were performed using SPSS version 23.0 and statistical significance was accepted when *p* ≤ 0.05. Data are presented as mean ± SD. A small number of participants did not complete all tests due to faults during data capture or inaccessibility for the specific tests.

## 3. Results

### 3.1. General Characteristics of Participants

The general characteristics of the participants are presented in Table 1.

### 3.2. Genotyping and SNP Associations with Skeletal Muscle Phenotypes

All SNPs were in Hardy–Weinberg equilibrium (*p* > 0.15) (Table S3) and the genotyping success rate was > 99%. Of the 24 SNPs analysed, 10 showed associations with skeletal muscle phenotypes. None of *ACTN3* rs1815739, *ACE* rs4341, *CNTFR* rs2070802, *IL6* rs1800795, *IGF1* rs35767, *ACVR1B* rs2854464, *ESR1* rs1999805, *ESR1* rs4870044, *ID3* rs11574, *MTHFR* rs1801131, *MTHFR* rs1537516, *MTHFR* rs17421511, *VDR* rs2228570 or *TTN* rs10497520 were associated with any of the skeletal muscle size and strength measures.

**Figure 1:**
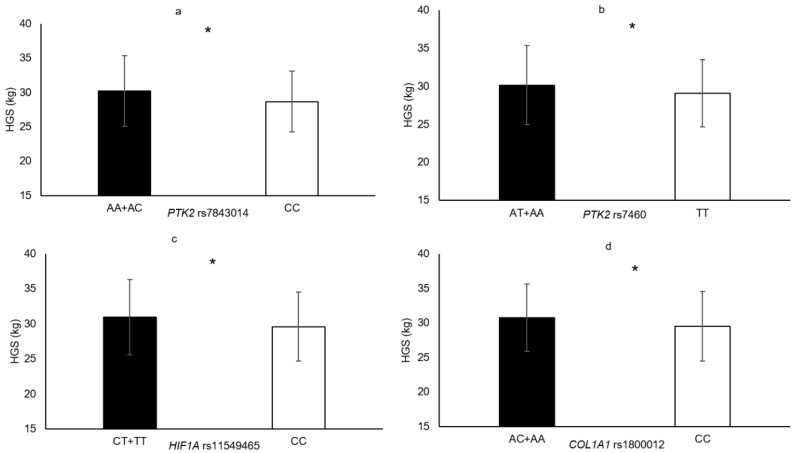
Handgrip strength in genotype groups for (**a**) *PTK2* rs7843014 (AA + AC = 247 vs. CC = 57, Δ = 5.3%); (**b**) *PTK2* rs7460 (TT = 72 vs. AT + AA = 233, Δ = 3.6%); (**c**) *HIF1A* rs11549465 (CT + TT = 64 vs. CC = 241, Δ = 4.6%); (**d**) *COL1A1* rs1800012 (AA + AC = 99 vs. CC = 205, Δ = 4.1%). * denotes significant difference. Data are mean ± SD.

**Figure 2:**
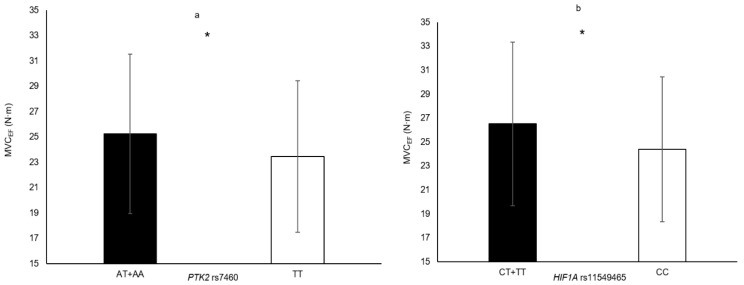
Isometric elbow flexion maximum voluntary contraction in genotype groups for (**a**) *PTK2* rs7460 (TT = 71 vs. AT + AA = 233, Δ = 7.7%); (**b**) *HIF1A* rs11549465 (CT + TT = 63 vs. CC = 241, Δ = 8.7%). * denotes significant difference. Data are mean ± SD.

**Figure 3:**
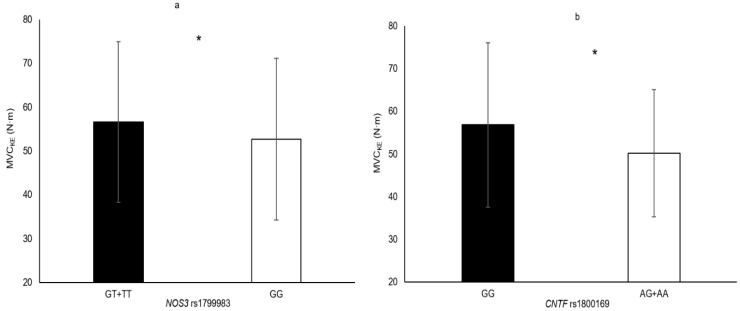
Isometric knee extension maximum voluntary contraction in genotype groups for (**a**) *NOS3* rs1799983 (GG = 117 vs. GT + TT = 185, Δ = 7.5%); (**b**) *CNTF* rs18000169 (GG = 222 vs. AG + AA = 80, Δ = 13.2%). * denotes significant difference. Data are mean ± SD.

**Figure 4:**
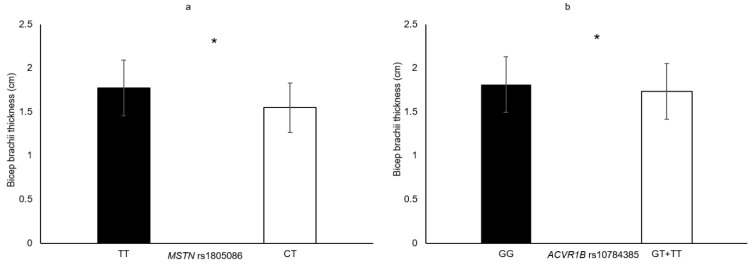
Biceps brachii thickness in genotype groups for (**a**) *ACVR1B* rs10784385 (GG = 124 vs. GT + TT = 166, Δ = 4.6%); (**b**) *MSTN* rs1805086 (CT = 9 vs. TT = 282, Δ = 14.7%). * denotes significant difference. Data are mean ± SD.

**Figure 5:**
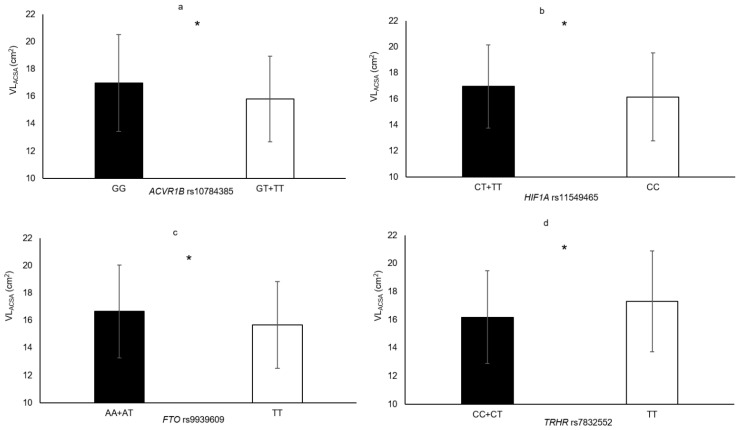
Vastus lateralis muscle cross sectional area in genotype groups for (**a**) *ACVR1B* rs10784385 (GT + TT = 168 vs. GG = 121, Δ = 7.3%); (**b**) *HIF1A* rs11549465 (CC = 228 vs. CT + TT = 62, Δ = 5.0%); (**c**) *FTO* rs9939609 (AA + AT = 188 vs. TT = 102, Δ = 6.2%); (**d**) *TRHR* rs7832552 (TT = 37 vs. CT + CC = 252, Δ = 7.0%). * denotes significant difference. Data are mean ± SD.

In the following section, only the SNPs associated with skeletal muscle phenotypes are presented. Genotype–muscle phenotype associations were observed in this sample of elderly women for the following: HGS (*PTK2* rs7843014, *PTK2* rs7460, *HIF1A* rs11549465 and *COL1A1* rs1800012; Figure 1), MVC_EF_ (*HIF1A* rs11549465 and *PTK2* rs7460; Figure 2), MVC_KE_ (*CNTF* rs1800169 and *NOS3* rs1799983; Figure 3), biceps brachii thickness (*MSTN* rs1805086 and *ACVR1B* rs10783485; Figure 4) and VL_ACSA_ (*TRHR* rs7832552, *ACVR1B* rs10783485, *HIF1A* rs11549465 and *FTO* rs9939609; Figure 5). Participants possessing the genotype associated with phenotypes for greater muscle size (biceps brachii thickness and VL_ACSA_) or strength (HGS, MVC_EF_ and MVC_KE_) were considered as having the favourable genotype. Elderly women in the favourable genotype groups were 3.6–13.2% stronger and had 4.6–14.7% larger muscle than their counterparts with less favourable genotypes (all *p* < 0.05, specific phenotypes shown in Table 2).

## 4. Discussion

The current study identified associations between several SNPs and skeletal muscle phenotypes related to muscle size (biceps brachii thickness and VL_ACSA_) and muscle strength (HGS, MVC_EF_ and MVC_KE_) in elderly women. The genetic variants associated with skeletal muscle phenotypes can be described by the biological roles of the genes. For the purpose of this discussion, the genes are grouped according to their potential role in terms of how they can affect muscle tissue: (1) structural proteins, (2) transcriptional regulators, (3) antagonists of muscle growth, (4) body composition regulators and (5) myotrophic factors.

### 4.1. Structural Proteins

*PTK2* rs7460 was associated with HGS and MVC_EF_, and *PTK2* rs7843014 and *COL1A1* rs1800012 were associated with HGS. These genes encode for a component of muscle structural proteins and the extracellular matrix (ECM) and thus might provide strength and integrity for the muscle fibre. For example, the *PTK2* rs7460 A-allele, identified here as a favourable allele, has been previously associated with higher baseline specific force in Caucasian men [42,43]. It has been speculated that *PTK2* rs7460 AA might favour more integrin-ECM bonds and result in a higher costamere density [43], which may favour lateral force transmission [42]. Higher gene expression has been observed with AA homozygotes compared to TT for *PTK2* rs7460 [44], which may result in an increase in the number of integrin ECM bonds compared to TT genotypes and thus higher lateral force transmission with higher resultant muscle strength in the present elderly population.

For *COL1A1* rs1800012, there is evidence that the A-allele of the Sp1-COL1A1 binding site polymorphism is linked with enhanced DNA-protein binding, encouraging transcription and elevated expression of *COL1A1* in osteoblast culture [45], with a higher proportion of collagen α1 compared to collagen α2. Despite contrasting data regarding bone health [46,47,48,49,50,51,52,53,54], in athletes the *COL1A1* A-allele seems to be protective for tendon and ligament injuries [55]. Collagen is predominant in tendons and tendon function is affected by the quality and architecture of collagen fibres [56,57]. It is therefore possible that there is some advantageous effect of the *COL1A1* rs1800012 A-allele that could protect from soft tissue injury, improve tendon function and contribute to the higher strength observed in elderly women.

### 4.2. Transcriptional Regulators

The present study has identified associations of transcription factor and transcription regulator gene variants *HIF1A* rs11549465 and *NOS3* rs1799983 with skeletal muscle phenotypes. These gene variants might change the transcription of genes affecting skeletal muscle formation and thus contribute to the variability in muscle size and strength in the elderly women. For instance, transcription factor HIF1A is the sub-unit of heterodimeric transcriptional factor HIF1, which induces the transcription of genes involved in cellular proliferation and survival [8,58], with the *HIF1A* rs11549465 T-allele associated with enhanced trans-activation capacity [59]. Enhanced transactivation capacity with the T-allele could be associated with increased cell proliferation and a higher proportion of fast-twitch fibres, which may explain the larger VL_ACSA_ and higher muscle strength (HGS and MVC_EF_) in the T-allele carriers in the present elderly population. In line with this, the cross-sectional area of type IIb muscle fibres was on average 16% larger in HIF-1α transfected compared to non-transfected extensor digitorum longus muscles in rats [60]. In humans, immuno-histochemical analysis of vastus lateralis muscle has shown a higher proportion of fast-twitch muscle fibres in a *HIF1A* rs11549465 T-allele (Ser) group compared to a C-allele (Pro) group; 13.8% in the Pro/Ser group compared to 8.2% in the Pro/Pro (CC) group [61]. This suggests that the T-allele could be favourable to powerful movements. Furthermore, previous studies have observed the T-allele to be more common in power-oriented athletes [62,63,64], and to be associated with maximal oxygen consumption post-training in older Caucasians [65] and young women [66].

*NOS3* encodes endothelial nitric oxide synthase (eNOS) which catalyses nitric oxide (NO) synthesis. NO plays a role in skeletal muscle fibre conversion [67], mitochondrial energy production [68] and muscle hypertrophy [69]. The physiological activities of skeletal muscle, such as excitation–contraction coupling, force generation, calcium homeostasis, metabolism and bioenergetics [70,71], are highly regulated by NO. Muscle atrophy in *NOS3* knockout mice [72,73,74] further implies the crucial role of NO in skeletal muscle growth. Despite the fact that higher NO activity has been associated with the *NOS3* rs1799983 G-allele [75,76], our present observation is in line with other studies reporting T-allele as beneficial for skeletal muscle function/performance. For instance, T-allele has been identified as favourable in some athletes [77,78], associated with adaptation to resistance training [79] and beneficial for maintaining normal muscle mass above the sarcopenic threshold [36].

### 4.3. Antagonists of Muscle Growth

In the elderly women analysed in our study, *ACVR1B* rs10783485 GG homozygotes had a thicker biceps brachii and larger VL_ACSA_ than T-allele carriers. The *ACVR1B* gene encodes the activin A receptor type 1b, which affects muscle growth negatively by stimulating the myostatin and activin signalling pathways [80,81]. Previous studies have reported the C-allele (G-allele in this case) as being favourable for higher knee strength [82] and an increment in rectus femoris diameter post-training in coronary artery disease patients [83]. The association of knee strength with *ACVR1B* rs10783485 was described by the considerable linkage disequilibrium (r^2^ = 0.15–0.44) between *ACVR1B* rs10783485 and *ACVR1B* rs2854464 [82]. The previous authors suggested that the observed association of *ACVR1B* rs2854464 with greater strength in the study could be due to higher affinity between the 3′ untranslated region of ACVR1B mRNA and microRNA-24, leading to more effective translational inhibition and decay of ACVR1B mRNA [82]. The pharmacological blockade of activin A signalling has been observed to increase muscle mass [84], so the present association with muscle size might be due to the decay of ACVR1B mRNA, enhancing muscle growth in GG homozygotes via the reduced activation of activin myostatin pathways.

The present study also identified *MSTN* rs1805086 TT (K-variant) homozygotes as the favourable genotype for thicker biceps brachii. K153 has been previously associated with higher muscle strength [85], muscle mass and functional capacity in women [86], as well as with better performance in high jumps [87] than the 153R variant; however, conflicting results do exist for skeletal muscle phenotypes [88,89]. K153R mutant (R-variant) increases the susceptibility of promyostatin for furin-cleavage [90], and thus could facilitate the formation of more latent myostatin [90]. Since myostatin is the negative regulator of muscle growth [80], the thicker biceps brachii in the present elderly women with TT (KK) homozygotes could be due to lower myostatin activity in the TT (KK) homozygotes compared to its mutant R-variant.

### 4.4. Body Composition Regulators

Body composition indices such as BMI, fat mass and other obesity-related phenotypes are strongly regulated by the *FTO* gene [91,92,93]. Impaired skeletal muscle development has been observed in FTO-deficient mice [94]. Furthermore, there is an increment in FTO expression during myogenic differentiation and FTO silencing leads to myogenic suppression [94]. Recent studies have found an association between FTO and appendicular lean mass, with a decrement in appendicular muscle mass when fat mass was controlled [95,96]. Our finding of an association of the *FTO* A-allele with larger VL muscle cross-sectional area in the present study is partly consistent with previous studies showing associations with parameters such as higher fat mass, lean body mass [91,92] and BMI [97,98,99]. However, we previously reported the A-allele to be associated with a greater risk of sarcopenia [36]. These apparent differences in genotypic associations could reflect fat infiltration [100,101,102] and the relative inability of older muscle to respond to loading [103].

### 4.5. Myotrophic Factors

*CNTF* rs18000169 and *TRHR* rs7832552 were associated with skeletal muscle phenotypes in the present study. CNTF is a signalling molecule with neurotrophic and myotrophic roles [104,105] and CNTF treatment results in enhanced myogenesis and diminished atrophy mediators [106]. Furthermore, CNTF level decreases with ageing and exogenous administration of CNTF in older rats has been shown to improve muscle strength [107]. A functional gene variant, *CNTF* rs1800169, with AA genotype produces a non-functional protein [108], and the present finding is consistent with most previous studies which report the GG genotype as the favourable genotype for skeletal muscle phenotypes [109,110,111]. CNTF α contributes to STAT3 activation [112], which has been linked with myoblast proliferation [113]. It is therefore possible that the elderly women with GG genotypes have functional proteins that could contribute to effective myogenesis, which is important for muscle maintenance, and hence are stronger than A-allele carriers.

TRHR stimulates the hypothalamic pituitary–thyroid axis, leading to the release of thyroxin. Thyroxin plays an important role in skeletal muscle development and the attenuation of age-related changes in tissue function [114], where reductions in thyroid hormone levels result in muscle weakness [115]. A genome-wide association study (GWAS) found that the *TRHR* rs7832552 TT genotype is associated with a higher lean body mass in US Caucasians [116] and the TT genotype also seems to be positively associated with sprint/power performance [117]. There was higher *TRHR* gene expression with T-allele in C2C12 skeletal muscle cell lines of mice [118]. It is possible, therefore, that TT genotype is associated with the optimal expression of thyroid hormone receptor and thus associated with favourable skeletal muscle phenotypes, such as VL_ACSA_ in the present elderly population.

### 4.6. Implications and Limitations

The present study has found genotype associations with a range of skeletal muscle phenotypes in elderly women. These genotype associations offer meaningful advantages for the measured skeletal muscle phenotypes; for instance, the presence of favourable SNPs is associated with higher muscle strength by 3.6–13.2%, which may well translate into an advantage for functional capacity. The measured ranges of benefits to muscle strength in the present study are similar to the positive adaptations that have previously been reported in elderly people after an exercise intervention [119,120]. The associations we report therefore seem to have real-world relevance.

No single gene variant was associated with all the measured muscle phenotypes, probably due to the modest influence of those specific gene variants on the specific muscle measures. Even more striking is the fact that 14 SNPs, contrary to our hypotheses and the suspected roles of these genes in muscle mass regulation, were not associated with any muscle phenotypes. The absence of associations may partly be attributable to the fact that we recruited independently living and recreationally active participants, with which the discriminating power of genotypes was not enough to distinguish the difference in muscle phenotypes. One should also not dismiss the role of redundancy, where tissues are able to cope with disadvantageous genotypes through other adaptations. We also suggest that the observed SNP–phenotype associations should be replicated in an independent elderly population to confirm our findings. The effect of SNPs on skeletal muscle phenotypes could perhaps be understood more holistically if a polygenic approach is adopted involving all SNPs, considering that muscle size and strength are polygenic in nature. In the elderly, however, only a limited number of SNPs have been associated with muscle phenotypes [10,86,121,122]; it is therefore necessary to first identify many SNPs before investigating their collective ability to capture the observed phenotypic variance, utilizing a polygenic model. Despite these potential shortcomings, the gene variants associated with skeletal muscle phenotypes in the present study could be beneficial in identifying those individuals most prone to muscle wasting conditions such as cachexia and sarcopenia.

## 5. Conclusions

The present study identified the association of ten gene variants (*HIF1A* rs11549465, *PTK2* rs7460, *ACVR1B* rs10783485, *PTK2* rs7843014, *COL1A1* rs1800012, *CNTF* rs1800169, *NOS3* rs1799983, *MSTN* rs1805086, *TRHR* rs7832552 and *FTO* rs9939609) and skeletal muscle phenotypes in an elderly population. The identification of gene variants associated with muscle size and strength measures might help in screening the population prone to sarcopenia in old age.

## Figures and Tables

**Table 1:** General characteristics of the participants.

Variables	Mean ± SD (*n* = 307)
Age (years)	71 ± 6
Mass (kg)	66.3 ± 11.3
Height (m)	1.60 ± 0.06
BMI (kg/m^2^)	25.9 ± 4.2
HGS (kg)	29.9 ± 5.0
MVC_EF_ (N·m)	24.8 ± 6.2
MVC_KE_ (N·m)	55.1± 18.4
Biceps brachii thickness (cm)	1.76 ± 0.32
VL_ACSA_ (cm^2^)	16.3 ± 3.4

Abbreviations: BMI, body mass index, HGS, handgrip strength, MVC_EF_, isometric elbow flexion maximal voluntary contraction, MVC_KE_, isometric knee extension maximal voluntary contraction, VL_ACSA_, vastus lateralis anatomical cross-sectional area.

**Table 2:** Associations between SNPs and skeletal muscle phenotypes in elderly Caucasian women.

Polymorphisms	Genotypes	Phenotypes	% Difference	*p*-Value
*TRHR* rs7832552	TT vs. CC+CT	VL_ACSA_	7.0%	0.036
*HIF1A* rs11549465	CT + TT vs. CC	VL_ACSA_ HGS MVC_EF_	5.0% 4.6% 8.7%	0.033 0.012 0.007
*PTK2* rs7460	AT + AA vs. TT	MVC_EF_ HGS	7.7% 3.6%	0.015 0.042
*PTK2* rs7843014	AC + AA vs. CC	HGS	5.3%	0.018
*ACVR1B* rs10783485	GT + TT vs. GG	VL_ACSA_ Biceps brachii thickness	7.3% 4.6%	0.009 0.045
*FTO* rs9939609	TT vs. AA + AT	VL_ACSA_	6.2%	0.014
*NOS3* rs1799983	TT + GT vs. GG	MVC_KE_	7.5%	0.042
*CNTF* rs1800169	AA + AG vs. GG	MVC_KE_	13.2%	0.004
*MSTN* rs1805086	CT vs. TT	Biceps brachii thickness	14.7%	0.035
*COL1A1* rs1800012	AA + AC vs. CC	HGS	4.1%	0.013

Grey shadings denote the favourable groups for skeletal muscle phenotypes. Abbreviations: HGS—handgrip strength, VL_ACSA_—vastus lateralis anatomical cross-sectional area, MVC_KE_—isometric knee extension torque maximal voluntary contraction, MVC_EF_—isometric elbow flexion torque maximal voluntary contraction.

## Supplementary Materials

The following are available online at https://www.mdpi.com/2073-4425/11/12/1459/s1, Table S1: Previous associations of single nucleotide polymorphisms/gene product and muscle-related phenotypes/performance; Table S2: Polymorphisms used in genotyping, identification of allele-specific probes and nucleotide reporting based on forward and reverse strand sequencing; Table S3: Genotype frequency and Hardy–Weinberg Equilibrium.

## Declaration

The present study forms the part of main author’s (P.K) thesis submitted in Manchester Metropolitan University, UK and KU Leuven, Belgium, for the fulfilment of joint Doctorate degree, with the thesis entitled “The genetics of sarcopenia and skeletal muscle phenotypes in elderly Caucasian women”.

## References

[1] Cruz-Jentoft A.J., Baeyens J.P., Bauer J.M., Boirie Y., Cederholm T., Landi F., Martin F.C., Michel J.-P., Rolland Y., Schneider S.M. (2010). Sarcopenia: European consensus on definition and diagnosisReport of the European Working Group on Sarcopenia in Older People. Age Ageing.

[2] Clark D.J., Patten C., Reid K.F., Carabello R.J., Phillips E.M., Fielding R.A. (2010). Impaired voluntary neuromuscular activation limits muscle power in mobility-limited older adults. J. Gerontol. Ser. A Biomed. Sci. Med. Sci..

[3] Reid K.F., Fielding R.A. (2012). Skeletal muscle power: A critical determinant of physical functioning in older adults. Exerc. Sport Sci. Rev..

[4] Abney M., McPeek M.S., Ober C. (2001). Broad and narrow heritabilities of quantitative traits in a founder population. Am. J. Hum. Genet..

[5] Silventoinen K., Magnusson P.K.E., Tynelius P., Kaprio J., Rasmussen F. (2008). Heritability of body size and muscle strength in young adulthood: A study of one million Swedish men. Genet. Epidemiol..

[6] Thomis M.A.I., Beunen G.P., Leemputte M.V., Maes H.H., Blimkie C.J., Claessens A.L., Marchal G., Willems E., Vlietinck R.F. (1998). Inheritance of static and dynamic arm strength and some of its determinants. Acta Physiol. Scand..

[7] Gao Y. (2010). The multiple actions of NO. Pflügers Arch. Eur. J. Physiol..

[8] Lee J.-W., Bae S.-H., Jeong J.-W., Kim S.-H., Kim K.-W. (2004). Hypoxia-inducible factor (HIF-1) α: Its protein stability and biological functions. Exp. Mol. Med..

[9] Wiener P., Woolliams J., Frank-Lawale A., Ryan M., Richardson R., Nute G., Wood J., Homer D., Williams J. (2009). The effects of a mutation in the myostatin gene on meat and carcass quality. Meat Sci..

[10] Charbonneau D.E., Hanson E.D., Ludlow A.T., Delmonico M.J., Hurley B.F., Roth S.M. (2008). ACE genotype and the muscle hypertrophic and strength responses to strength training. Med. Sci. Sports Exerc..

[11] Clarkson P.M., Devaney J.M., Gordish-Dressman H., Thompson P.D., Hubal M.J., Urso M., Price T.B., Angelopoulos T.J., Gordon P.M., Moyna N.M. (2005). ACTN3 genotype is associated with increases in muscle strength in response to resistance training in women. J. Appl. Physiol..

[12] Wakahara T., Takeshita K., Kato E., Miyatani M., Tanaka N.I., Kanehisa H., Kawakami Y., Fukunaga T. (2010). Variability of limb muscle size in young men. Am. J. Hum. Biol..

[13] Stebbings G.K., Morse C.I., Williams A.G., Day S.H. (2014). Variability and distribution of muscle strength and its determinants in humans. Muscle Nerve.

[14] Erskine R.M., Jones D.A., Maganaris C.N., Degens H. (2009). In vivo specific tension of the human quadriceps femoris muscle. Eur. J. Appl. Physiol..

[15] Garatachea N., Lucía A. (2013). Genes and the ageing muscle: A review on genetic association studies. Age.

[16] Tan L.-J., Liu S.-L., Lei S.-F., Papasian C.J., Deng H.-W. (2012). Molecular genetic studies of gene identification for sarcopenia. Hum. Genet..

[17] McCauley T., Mastana S.S., Folland J.P. (2010). ACE I/D and ACTN3 R/X polymorphisms and muscle function and muscularity of older Caucasian men. Eur. J. Appl. Physiol..

[18] Lexell J., Taylor C.C., Sjöström M. (1988). What is the cause of the ageing atrophy?: Total number, size and proportion of different fiber types studied in whole vastus lateralis muscle from 15- to 83-year-old men. J. Neurol. Sci..

[19] Janssen I., Heymsfield S.B., Ross R. (2002). Low relative skeletal muscle mass (sarcopenia) in older persons is associated with functional impairment and physical disability. J. Am. Geriatr. Soc..

[20] Martien S., Delecluse C., Boen F., Seghers J., Pelssers J., Van Hoecke A.-S., Van Roie E. (2015). Is knee extension strength a better predictor of functional performance than handgrip strength among older adults in three different settings?. Arch. Gerontol. Geriatr..

[21] Keller K., Engelhardt M. (2013). Strength and muscle mass loss with aging process. Age and strength loss. MusclesLigaments Tendons J..

[22] Janssen I., Heymsfield S.B., Wang Z., Ross R. (2000). Skeletal muscle mass and distribution in 468 men and women aged 18–88 yr. J. Appl. Physiol..

[23] McGrath R.P., Kraemer W.J., Al Snih S., Peterson M.D. (2018). Handgrip Strength and Health in Aging Adults. Sports Med..

[24] Bohannon R.W. (2015). Muscle strength: Clinical and prognostic value of hand-grip dynamometry. Curr. Opin. Clin. Nutr. Metab. Care.

[25] Stessman J., Rottenberg Y., Fischer M., Hammerman-Rozenberg A., Jacobs J.M. (2017). Handgrip strength in old and very old adults: Mood, cognition, function, and mortality. J. Am. Geriatr. Soc..

[26] Cho J., Lee I., Kang H. (2017). ACTN3 gene and susceptibility to sarcopenia and osteoporotic status in older Korean adults. Biomed. Res. Int..

[27] Romero-Blanco C., Artiga González M., Gómez-Cabello A., Vila-Maldonado S., Casajús J., Ara I., Aznar S. (2020). ACTN3 R577X polymorphism related to sarcopenia and physical fitness in active older women. Climacteric.

[28] He L., Khanal P., Morse C.I., Williams A., Thomis M. (2020). Associations of combined genetic and epigenetic scores with muscle size and muscle strength: A pilot study in older women. J. Cachexia Sarcopenia Muscle.

[29] Roberts H.C., Denison H.J., Martin H.J., Patel H.P., Syddall H., Cooper C., Sayer A.A. (2011). A review of the measurement of grip strength in clinical and epidemiological studies: Towards a standardised approach. Age Ageing.

[30] Villafañe J.H., Valdes K., Buraschi R., Martinelli M., Bissolotti L., Negrini S. (2016). Reliability of the handgrip strength test in elderly subjects with Parkinson Disease. Hand.

[31] Armatas V., Bassa E., Patikas D., Kitsas I., Zangelidis G., Kotzamanidis C. (2010). Neuromuscular differences between men and prepubescent boys during a peak isometric knee extension intermittent fatigue test. Pediatr. Exerc. Sci..

[32] Miyatani M., Kanehisa H., Ito M., Kawakami Y., Fukunaga T. (2004). The accuracy of volume estimates using ultrasound muscle thickness measurements in different muscle groups. Eur. J. Appl. Physiol..

[33] Ogasawara R., Thiebaud R.S., Loenneke J.P., Loftin M., Abe T. (2012). Time course for arm and chest muscle thickness changes following bench press training. Interv. Med. Appl. Sci..

[34] Morse C.I., Degens H., Jones D.A. (2007). The validity of estimating quadriceps volume from single MRI cross-sections in young men. Eur. J. Appl. Physiol..

[35] Reeves N.D., Maganaris C.N., Narici M.V. (2004). Ultrasonographic assessment of human skeletal muscle size. Eur. J. Appl. Physiol..

[36] Khanal P., He L., Stebbings G., Onambele-Pearson G.L., Degens H., Williams A., Thomis M., Morse C.I. (2020). Prevalence and association of single nucleotide polymorphisms with sarcopenia in older women depends on definition. Sci. Rep..

[37] He L., Khanal P., Morse C.I., Williams A., Thomis M. (2019). Differentially methylated gene patterns between age-matched sarcopenic and non-sarcopenic women. J. Cachexia Sarcopenia Muscle.

[38] Tintle N., Gordon D., Van Bruggen D., Finch S. (2009). The cost effectiveness of duplicate genotyping for testing genetic association. Ann. Hum. Genet..

[39] Fischer C.P., Plomgaard P., Hansen A.K., Pilegaard H., Saltin B., Pedersen B.K. (2004). Endurance training reduces the contraction-induced interleukin-6 mRNA expression in human skeletal muscle. Am. J. Physiol. Endocrinol. Metab..

[40] Benjamini Y., Hochberg Y. (1995). Controlling the false discovery rate: A practical and powerful approach to multiple testing. J. R. Stat. Soc. Ser. B.

[41] McDonald J. (2014). Handbook of Biological Statistics.

[42] Erskine R.M., Williams A.G., Jones D.A., Stewart C.E., Degens H. (2012). Do PTK2 gene polymorphisms contribute to the interindividual variability in muscle strength and the response to resistance training? A preliminary report. J. Appl. Physiol..

[43] Stebbings G.K., Williams A., Morse C., Day S. (2017). Polymorphisms in PTK2 are associated with skeletal muscle specific force: An independent replication study. Eur. J. Appl. Physiol..

[44] Garatachea N., Fuku N., He Z.-H., Tian Y., Arai Y., Abe Y., Murakami H., Miyachi M., Yvert T., Venturini L. (2014). PTK2 rs7460 and rs7843014 polymorphisms and exceptional longevity: A functional replication study. Rejuvenation Res..

[45] Mann V., Hobson E.E., Li B., Stewart T.L., Grant S.F.A., Robins S.P., Aspden R.M., Ralston S.H. (2001). A COL1A1 Sp1 binding site polymorphism predisposes to osteoporotic fracture by affecting bone density and quality. J. Clin. Investig..

[46] Garcia-Giralt N., Nogués X., Enjuanes A., Puig J., Mellibovsky L., Bay-Jensen A., Carreras R., Balcells S., Díez-Pérez A., Grinberg D. (2002). Two new single-nucleotide polymorphisms in the COL1A1 upstream regulatory region and their relationship to bone mineral density. J. Bone Miner. Res..

[47] Kostik M.M., Smirnov A.M., Demin G.S., Mnuskina M.M., Scheplyagina L.A., Larionova V.I. (2013). Genetic polymorphisms of collagen type I α1 chain (COL1A1) gene increase the frequency of low bone mineral density in the subgroup of children with juvenile idiopathic arthritis. EPMA J..

[48] Berg J.P., Lehmann E.H., Stakkestad J.A., Haug E., Halse J. (2000). The Sp1 binding site polymorphism in the collagen type I α 1 (COLIA1) gene is not associated with bone mineral density in healthy children, adolescents, and young adults. Eur. J. Endocrinol..

[49] Heegaard A.-M., Jørgensen H., Vestergaard A., Hassager C., Ralston S. (2000). Lack of influence of collagen type Iα1 Sp1 binding site polymorphism on the rate of bone loss in a cohort of postmenopausal Danish women followed for 18 years. Calcif. Tissue Int..

[50] Efstathiadou Z., Kranas V., Ioannidis J., Georgiou I., Tsatsoulis A. (2001). The Sp1 COLIA1 gene polymorphism, and not vitamin D receptor or estrogen receptor gene polymorphisms, determines bone mineral density in postmenopausal Greek women. Osteoporos. Int..

[51] Grant S.F., Reid D.M., Blake G., Herd R., Fogelman I., Ralston S.H. (1996). Reduced bone density and osteoporosis associated with a polymorphic Sp1 binding site in the collagen type I α 1 gene. Nat. Genet..

[52] Ji G., Yao M., Sun C., Zhang L., Han Z. (2009). Association of collagen type I α1 (COLIA1) Sp1 polymorphism with osteoporotic fracture in Caucasian post-menopausal women: A meta-analysis. J. Int. Med. Res..

[53] Lakatos P.L., Bajnok E., Tornai I., Folhoffer A., Horvath A., Lakatos P., Habior A., Szalay F. (2004). Insulin-like growth factor I gene microsatellite repeat, collagen type Iα1 gene Sp1 polymorphism, and bone disease in primary biliary cirrhosis. Eur. J. Gastroenterol. Hepatol..

[54] Rojano-Mejía D., Coral-Vázquez R.M., Espinosa L.C., López-Medina G., Aguirre-García M.C., Coronel A., Canto P. (2013). JAG1 and COL1A1 polymorphisms and haplotypes in relation to bone mineral density variations in postmenopausal Mexican-Mestizo Women. Age.

[55] Wang C., Li H., Chen K., Wu B., Liu H. (2017). Association of polymorphisms rs1800012 in COL1A1 with sports-related tendon and ligament injuries: A meta-analysis. Oncotarget.

[56] Franchi M., Trirè A., Quaranta M., Orsini E., Ottani V. (2007). Collagen structure of tendon relates to function. Sci. World J..

[57] Starkey C.P., Geesink G.H., Oddy V.H., Hopkins D.L. (2015). Explaining the variation in lamb longissimus shear force across and within ageing periods using protein degradation, sarcomere length and collagen characteristics. Meat Sci..

[58] Hashimoto T., Shibasaki F. (2015). Hypoxia-inducible factor as an angiogenic master switch. Front. Pediatr..

[59] Tanimoto K., Yoshiga K., Eguchi H., Kaneyasu M., Ukon K., Kumazaki T., Oue N., Yasui W., Imai K., Nakachi K. (2003). Hypoxia-inducible factor-1α polymorphisms associated with enhanced transactivation capacity, implying clinical significance. Carcinogenesis.

[60] Lunde I.G., Anton S.L., Bruusgaard J.C., Rana Z.A., Ellefsen S., Gundersen K. (2011). Hypoxia inducible factor 1α links fast-patterned muscle activity and fast muscle phenotype in rats. J. Physiol..

[61] Ahmetov I., Hakimullina A., Lyubaeva E., Vinogradova O., Rogozkin V. (2008). Effect of HIF1A gene polymorphism on human muscle performance. Bull. Exp. Biol..

[62] Gabbasov R.T., Arkhipova A.A., Borisova A.V., Hakimullina A.M., Kuznetsova A.V., Williams A.G., Day S.H., Ahmetov I.I. (2013). The HIF1A gene Pro582Ser polymorphism in Russian strength athletes. J. Strength Cond. Res..

[63] Cięszczyk P., Eider J., Arczewska A., Ostanek M., Leońska-Duniec A., Sawczyn S., Ficek K., Jascaniene N., Kotarska K., Sygit K. (2011). The HIF1A gene Pro582Ser polymorphism in polish power-orientated athletes. Biol. Sport.

[64] Drozdovska S.B., Dosenko V.E., Ahmetov I.I., Ilyin V.N. (2013). The association of gene polymorphisms with athlete status in Ukrainians. Biol. Sport.

[65] Prior S.J., Hagberg J.M., Phares D.A., Brown M.D., Fairfull L., Ferrell R.E., Roth S.M. (2003). Sequence variation in hypoxia-inducible factor 1α (HIF1A): Association with maximal oxygen consumption. Physiol. Genom..

[66] McPhee J.S., Perez-Schindler J., Degens H., Tomlinson D., Hennis P., Baar K., Williams A.G.E. (2011). HIF1A P582S gene association with endurance training responses in young women. Eur. J. Appl. Physiol..

[67] Martins K.J.B., St-Louis M., Murdoch G.K., MacLean I.M., McDonald P., Dixon W.T., Putman C.T., Michel R.N. (2012). Nitric oxide synthase inhibition prevents activity-induced calcineurin–NFATc1 signalling and fast-to-slow skeletal muscle fibre type conversions. J. Physiol..

[68] Brown G.C. (2007). Mechanisms of Inflammatory Neurodegeneration: iNOS and NADPH Oxidase.

[69] Smith L.W., Smith J.D., Criswell D.S. (2002). Involvement of nitric oxide synthase in skeletal muscle adaptation to chronic overload. J. Appl. Physiol..

[70] Eu J.P., Hare J.M., Hess D.T., Skaf M., Sun J., Cardenas-Navina I., Sun Q.-A., Dewhirst M., Meissner G., Stamler J.S. (2003). Concerted regulation of skeletal muscle contractility by oxygen tension and endogenous nitric oxide. Proc. Natl. Acad. Sci. USA.

[71] McConell G.K., Rattigan S., Lee-Young R.S., Wadley G.D., Merry T.L. (2012). Skeletal muscle nitric oxide signaling and exercise: A focus on glucose metabolism. Am. J. Physiol. Endocrinol. Metab..

[72] De Palma C., Morisi F., Pambianco S., Assi E., Touvier T., Russo S., Perrotta C., Romanello V., Carnio S., Cappello V. (2014). Deficient nitric oxide signalling impairs skeletal muscle growth and performance: Involvement of mitochondrial dysregulation. Skelet. Muscle.

[73] Sandri M., Coletto L., Grumati P., Bonaldo P. (2013). Misregulation of autophagy and protein degradation systems in myopathies and muscular dystrophies. J. Cell Sci..

[74] Sandri M. (2010). Autophagy in skeletal muscle. FEBS Lett..

[75] Persu A., Stoenoiu M.S., Messiaen T., Davila S., Robino C., El-Khattabi O., Mourad M., Horie S., Feron O., Balligand J.L. (2002). Modifier effect of ENOS in autosomal dominant polycystic kidney disease. Hum. Mol. Genet..

[76] Tesauro M., Thompson W.C., Rogliani P., Qi L., Chaudhary P.P., Moss J. (2000). Intracellular processing of endothelial nitric oxide synthase isoforms associated with differences in severity of cardiopulmonary diseases: Cleavage of proteins with aspartate vs. glutamate at position 298. Proc. Natl. Acad. Sci. USA.

[77] Zmijewski P., Cieszczyk P., Ahmetov I.I. (2018). The NOS3 G894T (rs1799983) and-786T/C (rs2070744) polymorphisms are associated with elite swimmer status. Biol. Sport.

[78] Weyerstraß J., Stewart K., Wesselius A., Zeegers M. (2018). Nine genetic polymorphisms associated with power athlete status–a meta-analysis. J. Sci. Med. Sport.

[79] Guidry M.A., Kostek M.A., Angelopoulos T.J., Clarkson P.M., Gordon P.M., Moyna N.M., Visich P.S., Zoeller R.F., Thompson P.D., Devaney J.M. (2012). Endothelial Nitric Oxide Synthase (NOS3) +894 G>T associates with physical activity and muscle performance among young adults. ISRN Vasc. Med..

[80] McPherron A.C., Lawler A.M., Lee S.-J. (1997). Regulation of skeletal muscle mass in mice by a new TGF-p superfamily member. Nature.

[81] Thomas M., Langley B., Berry C., Sharma M., Kirk S., Bass J., Kambadur R. (2000). Myostatin, a negative regulator of muscle growth, functions by inhibiting myoblast proliferation. J. Biol. Chem..

[82] Windelinckx A., De Mars G., Huygens W., Peeters M.W., Vincent B., Wijmenga C., Lambrechts D., Delecluse C., Roth S.M., Metter E.J. (2011). Comprehensive fine mapping of chr12q12-14 and follow-up replication identify activin receptor 1B (ACVR1B) as a muscle strength gene. Eur. J. Hum. Genet..

[83] Thomaes T., Thomis M., Onkelinx S., Goetschalckx K., Fagard R., Lambrechts D., Vanhees L. (2013). Genetic predisposition scores associate with muscular strength, size, and trainability. Med. Sci. Sports Exerc..

[84] Chen J.L., Walton K.L., Al-Musawi S.L., Kelly E.K., Qian H., La M., Lu L., Lovrecz G., Ziemann M., Lazarus R. (2015). Development of novel activin-targeted therapeutics. Mol. Ther..

[85] Seibert M.J., Xue Q.L., Fried L.P., Walston J.D. (2001). Polymorphic variation in the human myostatin (GDF-8) gene and association with strength measures in the Women’s Health and Aging Study II cohort. J. Am. Geriatr. Soc..

[86] González-Freire M., Rodríguez-Romo G., Santiago C., Bustamante-Ara N., Yvert T., Gómez-Gallego F., Rexach J.A.S., Ruiz J.R., Lucia A. (2010). The K153R variant in the myostatin gene and sarcopenia at the end of the human lifespan. Age.

[87] Santiago C., Ruiz J.R., Rodríguez-Romo G., Fiuza-Luces C., Yvert T., Gonzalez-Freire M., Gómez-Gallego F., Morán M., Lucia A. (2011). The K153R polymorphism in the myostatin gene and muscle power phenotypes in young, non-athletic men. PLoS ONE.

[88] Ivey F.M., Roth S.M., Ferrell R.E., Tracy B.L., Lemmer J.T., Hurlbut D.E., Martel G.F., Siegel E.L., Fozard J.L., Metter E.J. (2000). Effects of age, gender, and myostatin genotype on the hypertrophic response to heavy resistance strength training. J. Gerontol. Ser. A Biol. Sci. Med. Sci..

[89] Li X., Wang S.-J., Tan S.C., Chew P.L., Liu L., Wang L., Wen L., Ma L. (2014). The A55T and K153R polymorphisms of MSTN gene are associated with the strength training-induced muscle hypertrophy among Han Chinese men. J. Sports Sci..

[90] Szláma G., Trexler M., Buday L., Patthy L. (2015). K153R polymorphism in myostatin gene increases the rate of promyostatin activation by furin. FEBS Lett..

[91] Sonestedt E., Gullberg B., Ericson U., Wirfält E., Hedblad B., Orho-Melander M. (2011). Association between fat intake, physical activity and mortality depending on genetic variation in FTO. Int. J. Obes..

[92] Livshits G., Malkin I., Moayyeri A., Spector T.D., Hammond C.J. (2012). Association of FTO gene variants with body composition in UK twins. Ann. Hum. Genet..

[93] Frayling T.M., Timpson N.J., Weedon M.N., Zeggini E., Freathy R.M., Lindgren C.M., Perry J.R.B., Elliott K.S., Lango H., Rayner N.W. (2007). A common variant in the FTO gene is associated with body mass index and predisposes to childhood and adult obesity. Science.

[94] Wang X., Huang N., Yang M., Wei D., Tai H., Han X., Gong H., Zhou J., Qin J., Wei X. (2017). FTO is required for myogenesis by positively regulating mTOR-PGC-1α pathway-mediated mitochondria biogenesis. Cell Death Dis..

[95] Cordero A.I.H., Gregory J.S., Douglas A., Lionikas A. (2018). Genome-wide analysis in UK Biobank identifies over 100 QTLs associated with muscle mass variability in middle age individuals. bioRxiv.

[96] Zillikens M.C., Demissie S., Hsu Y.-H., Yerges-Armstrong L.M., Chou W.-C., Stolk L., Livshits G., Broer L., Johnson T., Koller D.L. (2017). Large meta-analysis of genome-wide association studies identifies five loci for lean body mass. Nat. Commun..

[97] Jacobsson J.A., Schiöth H.B., Fredriksson R. (2012). The impact of intronic single nucleotide polymorphisms and ethnic diversity for studies on the obesity gene FTO. Obes. Rev..

[98] Al-Serri A., Al-Bustan S.A., Kamkar M., Thomas D., Alsmadi O., Al-Temaimi R., Mojiminiyi O.A., Abdella N.A. (2018). Association of FTO rs9939609 with Obesity in the Kuwaiti Population: A Public Health Concern?. Med. Princ. Pract..

[99] Heffernan S.M., Stebbings G., Kilduff L.P., Erskine R., Day S.H., Morse C., McPhee J., Cook C., Vance B., Ribbans W.J. (2017). Fat mass and obesity associated (FTO) gene influences skeletal muscle phenotypes in non-resistance trained males and elite rugby playing position. BMC Genet..

[100] Borkan G.A., Hults D.E., Gerzof S.G., Robbins A.H., Silbert C.K. (1983). Age changes in body composition revealed by computed tomography. J. Gerontol..

[101] Visser M., Goodpaster B.H., Kritchevsky S.B., Newman A.B., Nevitt M., Rubin S.M., Simonsick E.M., Harris T.B. (2005). Muscle mass, muscle strength, and muscle fat infiltration as predictors of incident mobility limitations in well-functioning older persons. J. Gerontol. Ser. A Biol. Sci. Med. Sci..

[102] Delmonico M.J., Harris T.B., Visser M., Park S.W., Conroy M.B., Velasquez-Mieyer P., Boudreau R., Manini T.M., Nevitt M., Newman A.B. (2009). Longitudinal study of muscle strength, quality, and adipose tissue infiltration. Am. J. Clin. Nutr..

[103] Tomlinson D.J., Erskine R., Winwood K., Morse C., Onambélé G. (2014). The impact of obesity on skeletal muscle architecture in untrained young vs. old women. J. Anat..

[104] Forger N.G., Roberts S.L., Wong V., Breedlove S.M. (1993). Ciliary neurotrophic factor maintains motoneurons and their target muscles in developing rats. J. Neurosci..

[105] Ip N.Y., McClain J., Barrezueta N.X., Aldrich T.H., Pan L., Li Y., Wiegand S.J., Friedman B., Davis S., Yancopoulos G.D. (1993). The α component of the CNTF receptor is required for signaling and defines potential CNTF targets in the adult and during development. Neuron.

[106] Tsompanidis A., Vafiadaki E., Blüher S., Kalozoumi G., Sanoudou D., Mantzoros C.S. (2016). Ciliary neurotrophic factor upregulates follistatin and Pak1, causes overexpression of muscle differentiation related genes and downregulation of established atrophy mediators in skeletal muscle. Metabolism.

[107] Guillet C., Auguste P., Mayo W., Kreher P., Gascan H. (1999). Ciliary neurotrophic factor is a regulator of muscular strength in aging. J. Neurosci..

[108] Takahashi R., Yokoji H., Misawa H., Hayashi M., Hu J., Deguchi T. (1994). A null mutation in the human CNTF gene is not causally related to neurological diseases. Nat. Genet..

[109] Roth S.M., Schrager M.A., Ferrell R.E., Riechman S.E., Metter E.J., Lynch N.A., Lindle R.S., Hurley B.F. (2001). CNTF genotype is associated with muscular strength and quality in humans across the adult age span. J. Appl. Physiol..

[110] Arking D.E., Fallin D.M., Fried L.P., Li T., Beamer B.A., Xue Q.L., Chakravarti A., Walston J. (2006). Variation in the ciliary neurotrophic factor gene and muscle strength in older Caucasian women. J. Am. Geriatr. Soc..

[111] Walsh S., Kelsey B.K., Angelopoulos T.J., Clarkson P.M., Gordon P.M., Moyna N.M., Visich P.S., Zoeller R.F., Seip R.L., Bilbie S. (2009). CNTF 1357 G→ A polymorphism and the muscle strength response to resistance training. J. Appl. Physiol..

[112] Lee N., Spearry R.P., Rydyznski C.E., MacLennan A.J. (2019). Muscle ciliary neurotrophic factor receptor α contributes to motor neuron STAT 3 activation following peripheral nerve lesion. Eur. J. Neurosci..

[113] Zhang C., Li Y., Wu Y., Wang L., Wang X., Du J. (2013). Interleukin-6/signal transducer and activator of transcription 3 (STAT3) pathway is essential for macrophage infiltration and myoblast proliferation during muscle regeneration. J. Biol. Chem..

[114] Larsson L., Li X., Teresi A., Salviati G. (1994). Effects of thyroid hormone on fast-and slow-twitch skeletal muscles in young and old rats. J. Physiol..

[115] Salvatore D., Simonides W.S., Dentice M., Zavacki A.M., Larsen P.R. (2014). Thyroid hormones and skeletal muscle—New insights and potential implications. Nat. Rev. Endocrinol..

[116] Liu X.-G., Tan L.-J., Lei S.-F., Liu Y.-J., Shen H., Wang L., Yan H., Guo Y.-F., Xiong D.-H., Chen X.-D. (2009). Genome-wide association and replication studies identified TRHR as an important gene for lean body mass. Am. J. Hum. Genet..

[117] Miyamoto-Mikami E., Murakami H., Tsuchie H., Takahashi H., Ohiwa N., Miyachi M., Kawahara T., Fuku N. (2017). Lack of association between genotype score and sprint/power performance in the Japanese population. J. Sci. Med. Sport.

[118] Fuku N., He Z.-H., Sanchis-Gomar F., Pareja-Galeano H., Tian Y., Arai Y., Abe Y., Murakami H., Miyachi M., Zempo H. (2015). Exceptional longevity and muscle and fitness related genotypes: A functional in vitro analysis and case-control association replication study with SNPs THRH rs7832552, IL6 rs1800795, and ACSL1 rs6552828. Front. Aging Neurosci..

[119] Cadore E.L., Casas-Herrero A., Zambom-Ferraresi F., Idoate F., Millor N., Gómez M., Rodriguez-Mañas L., Izquierdo M. (2014). Multicomponent exercises including muscle power training enhance muscle mass, power output, and functional outcomes in institutionalized frail nonagenarians. Age.

[120] Santos L., Ribeiro A.S., Schoenfeld B.J., Nascimento M.A., Tomeleri C.M., Souza M.F., Pina F.L., Cyrino E.S. (2017). The improvement in walking speed induced by resistance training is associated with increased muscular strength but not skeletal muscle mass in older women. Eur. J. Sport Sci..

[121] Ma T., Lu D., Zhu Y.-S., Chu X.-F., Wang Y., Shi G.-P., Wang Z.-D., Yu L., Jiang X.-Y., Wang X.-F. (2018). ACTN3 genotype and physical function and frailty in an elderly Chinese population: The Rugao Longevity and Ageing Study. Age Ageing.

[122] Kostek M.C., Devaney J.M., Gordish-Dressman H., Harris T.B., Thompson P.D., Clarkson P.M., Angelopoulos T.J., Gordon P.M., Moyna N.M., Pescatello L.S. (2010). A polymorphism near IGF1 is associated with body composition and muscle function in women from the Health, Aging, and Body Composition Study. Eur. J. Appl. Physiol..

