# Supplementary material for: The Association of Multiple Gene Variants with Ageing Skeletal Muscle Phenotypes in Elderly Women
Source: Genes (Basel). 2020 Dec 5;11(12):1459. doi: 10.3390/genes11121459 (PMC7762041; doi:10.3390/genes11121459)
Supplement: Supplementary file 1 [file genes-11-01459-s001.zip › genes-1000023-supply/supplementary tables/Table S2. Identification of genotypes.docx]

Table S2. Polymorphisms used in genotyping, identification of allele-specific probes, and nucleotide reporting based on forward and reverse strand

| SNPs | VIC® | FAM® | Forward strand  (5’-3’) | Reverse strand  (5’-3’) | |
| --- | --- | --- | --- | --- | --- |
| *ACTN3* rs1815739 | T-allele | C-allele | CTGAC[T/C]GAGAG |  | |
| *ACE* rs4341 | C-allele | G-allele | TCAAG[C/G]CATTC |  | |
| *CNTF* rs1800169 | A-allele | G-allele | TCCTC[A/G]GCCAG |  |  |
| *MSTN* rs1805086 | C-allele | T-allele | GGGCC[C/T]TTACT |  | |
| *COL1A1* rs1800012 | A-allele | C-allele | CGCCC[A/C]CATTC |  | |
| *VDR* rs2228570 | A-allele | G-allele | CCTCC[A/G]TCCCT |  | |
| *TRHR* rs7832552 | C-allele | T-allele | AGGTA[C/T]GAATG |  | |
| *PTK2* rs7843014 | A-allele | C-allele | ACCTA[A/C]GAAGT |  | |
| *PTK2* rs7460 | A-allele | T-allele |  | CAATG[T/A]TATTT | |
| *IGF1* rs35767 | A-allele | G-allele | TTTCC[A/G]CATGA |  | |
| *IL6* rs1800795 | C-allele | G-allele | CTTGC[C/G]ATGCT |  | |
| *ACVR1B* rs2854464 | A-allele | G-allele | CAGCC[A/G]TGGGA |  | |
| *ACVR1B* rs10783485 | G-allele | T-allele | CCGCT[G/T]TGATA |  | |
| *ESR1* rs1999805 | A-allele | G-allele | ATTGT[A/G]AATGC |  | |
| *ESR1* rs4870044 | C-allele | T-allele | GGAGA[C/T]GTTGA |  | |
| *MTHFR* rs1537516 | A-allele | G-allele | TCCTA[A/G]GCGGG |  | |
| *MTHFR* rs17421511 | A-allele | G-allele | CTGGA[A/G]TCTGA |  | |
| *MTHFR* rs1801131 | G-allele | T-allele | CACTT[G/T]CTTCA |  | |
| *HIF1A* rs11549465 | C-allele | T-allele | TGTCA[C/T]CATTA |  | |
| *ID3* rs11574 | C-allele | T-allele | CGGAG[C/T]GAGCT |  | |
| *NOS3* rs1799983 | G-allele | T-allele | GATGA[G/T]CCCCC |  | |
| *FTO* rs9939609 | A-allele | T-allele | AATTT[A/T]GTGAT |  | |
| *CNTFR* rs2070802 | A-allele | T-allele | TAATC[A/T]CTGGC |  | |
| *TTN* rs10497520 | C-allele | T-allele | AACTT[C/T]AGGTT |  | |
