# Supplementary material for: The Association of Multiple Gene Variants with Ageing Skeletal Muscle Phenotypes in Elderly Women
Source: Genes (Basel). 2020 Dec 5;11(12):1459. doi: 10.3390/genes11121459 (PMC7762041; doi:10.3390/genes11121459)
Supplement: Supplementary file 1 [file genes-11-01459-s001.zip › genes-1000023-supply/supplementary tables/Table S3. Genotype frequency and Hardy Weinberg test.docx]

Table S3 Genotype frequency and Hardy Weinberg Equilibrium

| SNPs | Frequency | χ² p-value | |
| --- | --- | --- | --- |
| *IL6* rs1800795  CC (n=61)  CG (n=145)  GG (n=99) | 20.0%  47.5%  32.5% | 0.356 | 0.837 |
| *FTO* rs9939609  AA (n=48)  AT (n=151)  TT (n=106) | 15.7%  49.5%  34.8% | 0.228 | 0.892 |
| *MTHFR* rs17421511  AA (n=7)  AG (n=78)  GG (n=220) | 2.3%  25.6%  72.1% | 0.001 | 0.99 |
| *ACVR1B* rs10783485  GG (n=128)  GT (n=147)  TT (n=29) | 42.1%  48.4%  9.5% | 2.036 | 0.361 |
| *NOS3* rs1799983  GG (n=118)  GT (n=144)  TT (n=43) | 38.7%  47.2%  14.1% | 0.008 | 0.996 |
| *ACVR1B* rs2854464  AA (n=153)  AG (n=128)  GG (n=24) | 50.2%  42.0%  7.8% | 0.150 | 0.928 |
| *PTK2* rs7460  TT (n=72)  AT (n=153)  AA (n=80) | 23.6%  50.2%  26.2% | 0.005 | 0.998 |
| *ESR1* rs1999805  AA (n=100)  AG (n=148)  GG (n=57) | 32.8%  48.5%  18.7% | 0.029 | 0.985 |
| *PTK2* rs7843014  AA ((n=105)  AC (n=142)  CC (n=57) | 34.5%  46.7%  18.8% | 0.534 | 0.766 |
| *VDR* rs2228570  AA (n=103)  AG (n= 154)  GG (n=48) | 33.8%  50.5%  15.7% | 0.585 | 0.747 |
| *ID3* rs11574  CC (n=178)  CT (n=111)  TT (n=16) | 58.4%  36.4%  5.2% | 0.059 | 0.971 |
| *CNTF* rs1800169  AA (n=3)  AG (n=77)  GG (n=225) | 1.0%  25.2%  73.8% | 1.662 | 0.436 |
| *ACE* rs4341  CC (n=63)  CG (n=142)  GG (n=100) | 20.7%  46.5%  32.8% | 0.921 | 0.631 |
| *CNTFR* rs2070802  AA (n=216)  AT (n=79)  TT (n=10) | 70.8%  25.9%  3.3% | 0.686 | 0.710 |
| *MTHFR* rs1801131  GG (n=27)  GT (n=133)  TT (n=144) | 8.9%  43.8%  47.3% | 0.224 | 0.894 |
| *ESR1* rs4870044  CC (n=158)  CT (n=122)  TT (n=24) | 52.0%  40.1%  7.9% | 0.004 | 0.998 |
| *COL1A1* rs1800012  AA (n=10)  AC (n=89)  CC (n=205) | 3.3%  29.3%  67.4% | 0.008 | 0.996 |
| *ACTN3* rs1815739  CC (n=103)  CT (n=133)  TT (n=68) | 33.9%  43.8%  22.4% | 3.899 | 0.142 |
| *HIF1A* rs11549465  CC (n=241)  CT (n=62)  TT (n=2) | 79.0%  20.3%  0.7% | 0.869 | 0.648 |
| *MSTN* rs1805086  CC (n=0)  CT (n=10)  TT (n=295) | 0.0%  3.3%  96.7% | 0.085 | 0.959 |
| *MTHFR* rs1537516  AA (n=4)  AG (n=56)  GG (n=245) | 1.3%  18.4%  80.3% | 0.154 | 0.926 |
| *TTN* rs10497520  CC (n=235)  CT (n=66)  TT (n=4) | 77.1%  21.6%  1.3% | 0.069 | 0.966 |
| *TRHR* rs7832552  CC (n=137)  CT (n=130)  TT (n=37) | 45.0%  42.8%  12.2% | 0.510 | 0.775 |
| *IGF1* rs35767  AA (n=4)  AG (n=76)  GG (n=225) | 1.3%  24.9%  73.8% | 0.740 | 0.691 |
